# Evaluating the Spatial Accessibility and Distribution Balance of Multi-Level Medical Service Facilities

**DOI:** 10.3390/ijerph16071150

**Published:** 2019-03-30

**Authors:** Meihan Jin, Lu Liu, De Tong, Yongxi Gong, Yu Liu

**Affiliations:** 1 Laboratory for Urban Future, Peking University (Shenzhen), Shenzhen 518055, China; jmeihan@hotmail.com; 2 Shenzhen Key Laboratory of Urban Planning and Decision Making, Harbin Institute of Technology (Shenzhen), Shenzhen 518055, China; lulululu1243@163.com; 3 School of Architecture, Harbin Institute of Technology (Shenzhen), Shenzhen 518055, China; 4 School of urban planning and design, Peking University Shenzhen Graduate School, Shenzhen 518055, China; tongde@pkusz.edu.cn; 5 Institute of Remote Sensing and Geographical Information Systems, School of Earth and Space Sciences, Peking University, Beijing 100871, China; liuyu@urban.pku.edu.cn

**Keywords:** medical service facilities, spatial accessibility, hierarchical two-step floating catchment area method, distance attenuation

## Abstract

Public medical service facilities are among the most basic needs of the public and are directly related to residents’ health. The balanced development of medical service facilities is of great significance. Public medical service facilities can be divided into different levels according to their medical equipment, service catchment, and medical quality, which is very important but has been ignored for a long time in accessibility evaluations. In this research, based on the hospital and population datasets of Shenzhen, we propose a hierarchical two-step floating catchment area (H2SFCA) method to evaluate the spatial accessibility of public medical resources considering the factors at different levels of medical resources. In the proposed method, the spatial accessibility of each level of public medical service facilities are evaluated using different distance attenuation functions according to the medical service’s scope. In addition, a measurement is proposed to evaluate the equity of medical service facilities based on accessibility and population density distributions. To synthesize the general spatial accessibility and the distribution balance of public medical service facilities, we standardize the spatial accessibility of public medical service facilities at each level and then calculate the weighted sums of the accessibility of each level. The general spatial equity of public medical service facilities is also evaluated. The results show that the accessibility and distribution balance of medical resources performs dissimilarly at the three levels and can be discriminated within different regions of the city. The accessibility of citywide medical facilities in Shenzhen decreases from the city center to the suburban area in a radial pattern and the accessibility and distribution balance in the suburban areas needs improvement.

## 1. Introduction

Rapid economic and social development has led to a transition period in China in recent years, and the equalization of basic public services has received unprecedented attention. Equalization of basic public services refers to the government’s efforts to provide the basic public supplies and services needed to meet the fundamental requirements of daily life for residents. Equalization of public services also reflects the principles of fairness and justice, which directly influence the basic conditions of residents’ survival and development. Public service facilities are among the most important components in the normal operation of society and include social infrastructure such as education, medical care, and sports; these services are provided throughout the city to serve its residents [1]. Rational and balanced planning and development of public service facilities are essential to facilitate the integrated development of urban and rural regions [2]. The planning of public service facilities is closely connected to residents’ daily lives, as well as the optimization and adjustment of the urban spatial structure [3]. The rational allocation and layout of public facility land use in the city can lead to intensive conservation of land. As part of the public facilities, public health service facilities are directly related to vital issues of residents and provide people with medical treatment and health care.

In this research, the accessibility of medical facilities mainly refers to the convenience with which people who need to reach medical facilities can access to medical care from a given location through a certain means of transport, i.e., the cost of access to medical services. Spatial accessibility provides a summary measure of two important and related components of access; first, the volume of services provided relative to the population’s size, and second, the proximity of services provided relative to the location of the population [4]. The accessibility of medical facilities can be influenced by the condition of traffic networks (travel distance or time), the socioeconomic characteristics of consumers (such as race or ability to pay), and integrated selection strategies.

Public service facility accessibility has been a long-term concern for researchers that can be dated back to the 1950s [5], and it remains a high-priority topic now. Some major findings have been made through the efforts of numerous researchers. Haynes et al. used postal code locations and resident data to discover that travel time exhibits a positive linear relation with patient registration at health care facilities in eastern England [6]. A similar conclusion was made by Tanser et al.: travel time is a key factor in accessibility [7]. Langford and Higgs analyzed postal code and population data in the UK with GIS tools and concluded that the accessibility of medical service facilities depends on the alternative spatial representation of the population [8]. More recently, researchers have focused on identifying problems in medical service facility distribution to offer a valid solution for improving medical facility accessibility. The results of research on medical care accessibility in Belgium indicated that at different geographic scales of analysis, different methods should be applied to the measurement of medical service accessibility [9]. Researchers also pointed out that the inequity of health care accessibility remains a key issue to be solved worldwide, including China [10,11,12,13,14].

As for the methods applied, multiple approaches have been proposed to estimate spatial access to medical services. The models used in the measurement of spatial accessibility of medical services include provider-to-population ratios, distance to the nearest provider, the gravitational model, and the two-step floating catchment area method. The provider-to-population ratio is also considered the supply ratio; it is the most popular spatial accessibility measurement because it requires easy-to-access data and does not require GIS analysis [4,15,16]. The provider-to-population method is capable of comparing supply between large geopolitical units or service areas and is usually applied in policy analysis to define minimal standards of supply and to identify underserved areas [17,18,19]. Nevertheless, supply ratios bear certain limitations, including the exclusion of patient border crossings at small geographic scales [6] and a lack of distance or travel impedance measurements [20]. Distance to the nearest provider is also a popular measurement of spatial accessibility and is usually referred to as travel impedance or travel cost. It is usually used to measure the distance from a residence or a population center to the medical facility and is considered a good fit for the measurement of distance from rural areas; however, it is not an efficient measurement in high-density population areas [21]. The gravity model is a method combining the indicators of accessibility and availability, reflecting a decrease in attraction between supply and demand due to an increase in distance. Previous research has shown that the gravity model is able to provide more valid measures of accessibility to public service facilities than other models and is suitable for both urban and rural regions [12,22,23,24]. More recently, the method of two-step floating catchment area (2SFCA) was first proposed in the early 2000s and has been widely applied in spatial accessibility measurements in multiple fields, including retail allocation, land use planning and public service facility evaluation [15,16,25]. Furthermore, the 2SFCA method has been modified since then, and the modified 2SFCA method has been proven to be more efficient than the original method [13,26,27,28,29,30,31,32]. In addition, some approaches also analyzed the possible reasons leading to certain accessibility distributions [7,33,34,35]. Yet, this study mainly focuses on evaluation methods for medical facility accessibility when considering the hierarchy.

Previous research on the accessibility of medical service facilities has led to remarkable progress in methods, yet there remain issues in urban planning related to balanced medical service facility accessibility for residents. Public medical services can be divided into different levels according to its medical technology, medical equipment, serving scale, and medical quality, the last two of which should be addressed but which have been ignored for a long time in accessibility evaluations. Although it has been suggested that the accessibility of medical service facilities differs at different levels [9,36], there is still lack of research on categorizing and measuring medical service facility accessibility with a hierarchical perspective. Medical service facilities at different levels always serve distinct purposes, and their capacities and service range also vary dramatically. For filling in the blanks of accessibility measurements at multiple medical service facility levels, this research focuses on measuring public medical service facility accessibilities in a hierarchical perspective and assessing the equilibrium of medical service facility distribution. The main contributions of this work are two-fold. First, a hierarchical two-step floating catchment area (H2SFCA) method is proposed to measure medical service facility accessibility at multiple public medical service levels, with different levels of medical service accessibility calculated respectively and a general accessibility synthesized. Second, a method for evaluating the equilibrium between public medical service facility distribution and the demographic distribution is also introduced. Overall, a system of methodology for evaluating accessibility hierarchically and the facility distribution equilibrium is proposed for a better understanding of public medical service facilities at multiple levels. We use Shenzhen as the study area and investigate the accessibility of medical service facilities at different levels, from mesoscopic to macroscopic. Moreover, the population distribution and medical service facility distribution in the city are combined to evaluate the equity of medical services in Shenzhen City.

## 2. Study Area and Datasets

### 2.1. Study Area

We chose one of the metropolises in China, Shenzhen, as our study area because its ongoing economic development and large population result in a major transportation burden. Shenzhen is located in southern Guangdong Province in South China and is situated immediately north of Hong Kong. After the institution of the reform and opening-up policy in China in 1979, Shenzhen became the first special economic zone in China and started booming: it grew from a tiny fishing village into a massive modern city. The data from Shenzhen utilized in this research were collected in 2015. By the end of 2015, the population in Shenzhen had reached approximately 20 million, and the per capita income in Shenzhen was 4951 yuan, ranking fourth among cities in China.

Originally, Shenzhen City was considered to consist of two parts: the SEZ (Shenzhen economic zone) and the part outside the SEZ. While a clear separation between the SEZ and the area outside the SEZ no longer exists, the influence of the previous partition remains in multiple aspects, although economic development has maintained an extremely high pace.

Influenced by historical and objective factors, the equitable development of medical facilities in Shenzhen is lagging behind that of other Chinese super metropolises, and numerous problems exist, such as inadequate resources and an unbalanced distribution of facilities. Since the establishment of the SEZ in 1980, economic development has been booming under the guidance of the principle of “taking economic construction as the center” and has produced remarkable results. However, the rapid development of the economy has attracted an enormous population, and basic public service facilities, including public medical facilities, bear certain issues, such as resource shortages and uneven distribution. Moreover, administrators realize that the development of public service facilities is obviously lagging behind economic development and urban construction in Shenzhen. Having experienced nearly 40 years of rapid economic development, Shenzhen has built a solid economic foundation, and its social development is now in the transition stage: simple economic development has gradually shifted to coordinated economic and social development. At this particular moment, an important issue for basic public service facilities is the equal development of public medical facilities, which has long been a public concern. It is crucial to evaluate the accessibility of medical services in Shenzhen to understand the supply and demand status of public medical facilities precisely and comprehensively and to effectively improve the convenience of public access to health care.

### 2.2. Datasets

**Figure 1:**
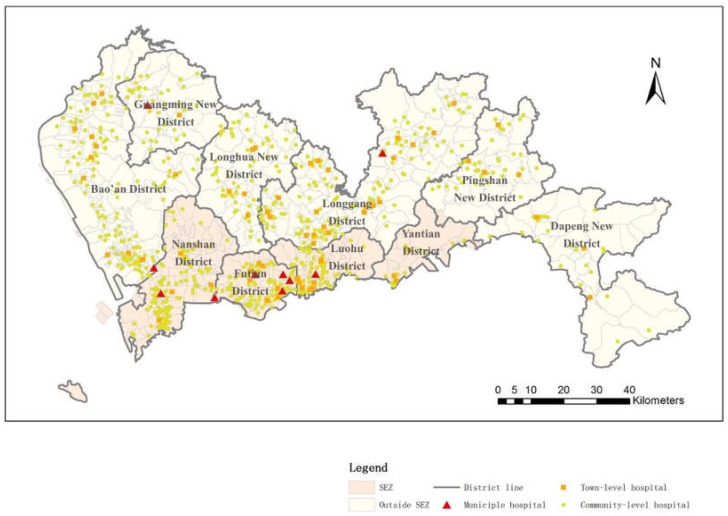
Spatial distribution of hospitals in Shenzhen.

In Shenzhen City, a major proportion of medical facilities consist of public medical facilities, and thus, in this research, public medical service facilities are the main focus. Generally, the medical service facilities in Shenzhen compose a comprehensive medical care system covering most kinds of medical departments and sections. From a hierarchical perspective, the medical facilities in Shenzhen can be categorized into three levels—namely, municipal hospitals, town-level hospitals and community-level hospitals—according to their different service scopes. The levels of medical resources have a great impact on residents’ medical treatments and serve as a strategy to balance the medical needs of residents; the higher-level medical facilities cover larger areas than the lower-level medical facilities, and the different levels serve different needs. For example, community hospitals mainly treat slight illnesses, whereas municipal hospitals treat vital diseases. The medical facility data used in this research are provided by the Urban Planning, Land and Resources of Shenzhen Municipality. This includes geographic locations, level categories, doctor numbers, nurse numbers, hospital bed numbers and address of all public hospitals in Shenzhen City. The distribution of three level hospitals are shown in Figure 1. There are 10 municipal hospitals in Shenzhen, and most are distributed throughout the SEZ. The district with the largest number of municipal hospitals is Futian District, with a total of five municipal hospitals. There are 68 town-level hospitals in Shenzhen City, and a total of 22 are inside the SEZ. Regarding community-level hospitals in Shenzhen, the total number is 686, among which 274 are located in the SEZ. Outside the SEZ, there are 412 community-level hospitals. The dataset of the patient numbers at different medical service facilities is offered by the Shenzhen Medical Information Center.

**Figure 2:**
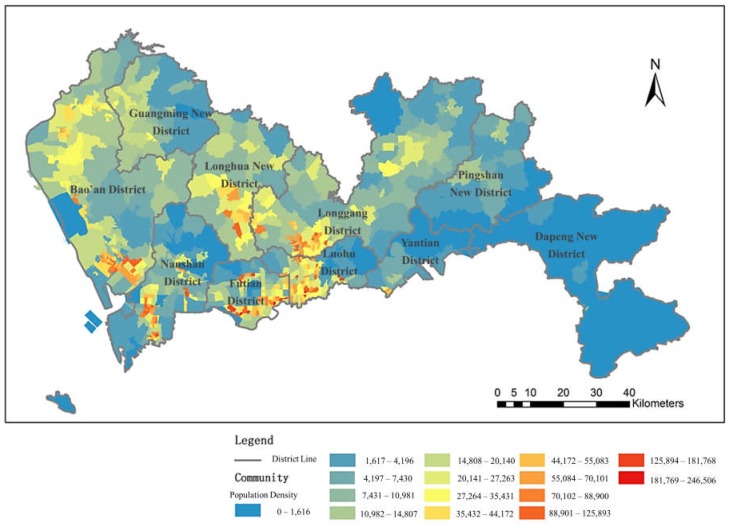
Distribution of population density in Shenzhen.

Another set of data utilized in this research is the population data of Shenzhen City, which was used to indicate the demand for medical facilities and was provided by Urban Planning, Land and Resources of Shenzhen Municipality. As shown in Figure 2, the population density is relatively high in Futian District, as well as in Luohu District, Longgang District and Longhua New District; the highest population density of the region is concentrated in Luohu District and Futian District. In addition, the population density is high in Bao’an District and Nanshan District.

The minimum spatial unit used in this research is the community scale. The community is the smallest area with completed functional facilities for residents’ basic needs in daily life and is also a population statistical unit recognized by the administration.

## 3. Methodology

This study aims to assess public medical service facility accessibility in a hierarchical perspective. The primary process is to categorize the medical service facilities in the city. The medical service data we obtained contains classifications of the hospitals and health care facilities in Shenzhen. In this study, we reorganize those classifications into three levels according the health care demands of the residents in the city into municipal hospitals, town-level hospitals and community-level hospitals. Community-level hospitals are defined as the preferential choice of medical service facility for patients who have minor illness with little cost for transportation, referring to community health care centers in this article particularly. Town-level hospitals are defined as healthcare facilities with advanced medical services that community-level facilities could not offer, referring to first-level or second-level hospitals. Municipal hospitals are the medical service facilities that are able to treat extremely severe diseases, referring to the third-level general hospital.

### 3.1. Accessibility Measurement

As introduced in the previous section, accessibility measurements of public service facilities have been thoroughly discussed in previous studies. The methods based on 2SFCA are the methods most commonly applied, and have been verified to be efficient and accurate for measuring the accessibility of public service facilities [15,36]. For the original 2SFCA, the procedure includes two steps. The first step is to calculate the supply-to-demand ratio within the catchment of each medical service facility, as defined by a threshold distance. The second step is to allocate the supplies that fall within the catchment of each demand region and sum up the supply-to-demand ratios calculated in the first step. The limitations of the traditional 2SFCA method are that there is no distance attenuation within the supply location service threshold and that there is no demand outside the service threshold [36]. Although several studies have extended the 2SFCA with distance attenuation functions, the hierarchy of medical service facilities is seldom taken in to consideration. Since medical service facilities at different levels should use different distance attenuations when calculating accessibility, we propose a hierarchical method (H2SFCA) based on 2SFCA to discriminate the distance attenuation for several levels of medical service facilities. 

First, for each supply location *j*, search all demand locations (*k*) that are within a threshold travel distance from location *j*, compute the supply-to-demand ratio *R_j_* within the catchment area, and consider the distance attenuation to convert [3]:

(1) $$R_{j}=S_{j}/\sum_{k}P_{k}f(dkj)$$

where *S_j_* is the capacity of supply at location *j*, the number of doctors in this study; *P_k_* is the demand at location *k*, the real population of the community in this study; *d_kj_* is the distance between *k* and *j*; and *f (d)* is the distance attenuation function.

Next, for each demand location *i*, search all supply locations *j* that are within the threshold distance from location *i* and sum up the supply-to-demand ratios *R_j_* that are converted to the distance attenuation at those locations to obtain the accessibility *A_i_* at demand location *i*:

(2) $$A_{i}=\sum_{j}R_{j}f(dij)=\sum_{j}\left[S_{j}f(dij)/\sum_{k}(P_{k}f(dkj)\right]$$

where *d_ij_* is the distance between *i* and *j* and *A_i_* is the medical accessibility of the demand location *i*; a larger value of *A_i_* indicates better accessibility at a location.

In this study, we assume that for different medical service facility levels, the radiation levels for the service should differ. Thus, the accessibility evaluation of medical service facilities should be calculated to determine the service level. In general, the distance attenuation function is defined as follows:

*f*(*d*) = *d*^−α^ (3)

where α is the attenuation coefficient in the case of distance attenuation α. For different levels of medical service facilities, the truncation distance and attenuation coefficient are also different. Truncation distance is the distance threshold indicating the maximum radius of the medical service range at a certain level. The attenuation coefficient stands for a measurement of distance decay speed. The larger the α is, the faster the distance decays. 

**Figure 3:**
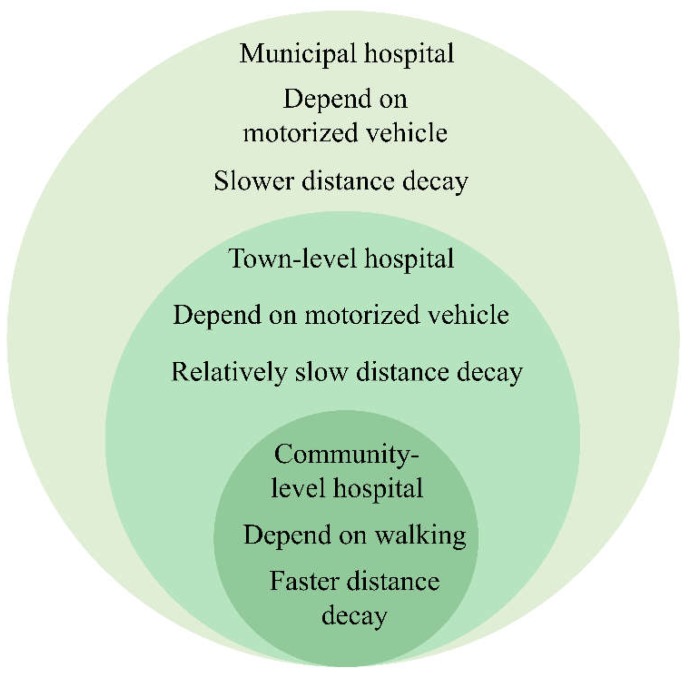
Schematic display of the attractive ranges for the three medical service levels in Shenzhen.

**Figure 4:**
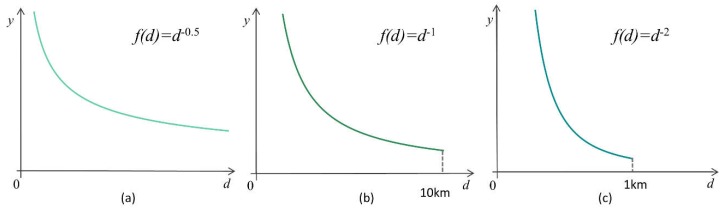
Schematic plot of distance attenuation functions for the three medical service levels in Shenzhen: (**a**) municipal hospital accessibility distance attenuation; (**b**) town-level hospital accessibility distance attenuation; (**c**) community-level hospital accessibility distance attenuation.

According to the previous research, the coefficient α is chosen based on travel distance, which is passively correlated with the travel distance. In previous research, α is sometimes valued as 0.6 for larger impact ranges, and 1–2 for minor ranges of influence [37,38]. The distance attenuation functions in this research are defined separately with varied attenuation coefficients and truncation distances for multiple levels of medical services according to their service range (Figure 3). A schematic plot graph for three distance attenuation functions is shown in Figure 4.

The government requires that community-level medical services are distributed within 15 to 20 min of access for residents [39]. Since the approximate average walking speed is 1.24 m/s in the city, the average walking distance within 30 min is 1488 m, defined as the truncation distance for community-level medical services. Early research has testified that for community-level medical services, a value of 2 is appropriate for the attenuation coefficient [40]. For town-level hospitals and municipal hospitals, no specific travel time is required. As residents with more serious medical cases will go to town-level hospitals for medical treatment and generally take a motor vehicle, the truncation distances are larger and the decay rates are slower than they are for community-level medical services. In this research, the truncation distance for town-level hospitals is 10,000 m, and the attenuation coefficient is 1. When residents are in a serious condition and are unable to be treated at community-level hospitals or town-level hospitals, residents will go to municipal hospitals for medical treatment. The service range of municipal hospitals covers the whole city. Therefore, for the municipal hospital service radius without truncation distance, the decay rate is also the slowest; in this study, it is set to 0.5 (Table 1).

Since residents demand different medical services from different levels of hospitals and since the attraction of the three hospital levels differs, resulting in different distance decay coefficients, the accessibility measurements of the three levels of hospitals are not quantitatively comparable. Despite the fact that the capacities and treatment scopes of different medical service levels differ from one another, each individual’s requirement for medical service is one package, including treatment from all three levels. Therefore, a synthesis of all public medical service accessibilities in general in the city is a crucial issue for supply and demand analyses of public medical services. For calculating medical service facility accessibility in general, first public medical service facility accessibilities at each level are normalized respectively using z-score normalization:

(4) $$z=\frac{x-\mu }{\sigma }$$

where *x* is the current sample value; *μ* is the average of the samples; *σ* is the standard deviation of the samples. As the treatment scopes of the three levels of medical services are different, the z-scores for each level of medical service accessibility are multiplied by a weight value according to the proportion of the total treatments for the corresponding medical service level. The general accessibility synthesized three levels of medical service facility accessibilities is therefore formalized as:

(5) $$G_{i}=w_{m}z_{m,i}+w_{t}z_{t,i}+w_{c}z_{c,i}$$

where *w_m_* is the weight of municipal hospital; *z_m,i_* is the z-score of the municipal hospital accessibility for unit *i*; *w_t_* is the weight of town-level hospital; *z_t,i_* is the z-score of the town-level hospital accessibility for unit *i*; *w_c_* is the weight of community-level hospital accessibility; *z_c,i_* is the z-score of the community-level hospital accessibility for unit *i*. 

### 3.2. Measurement of the Medical Service Distribution Balance

**Figure 5:**
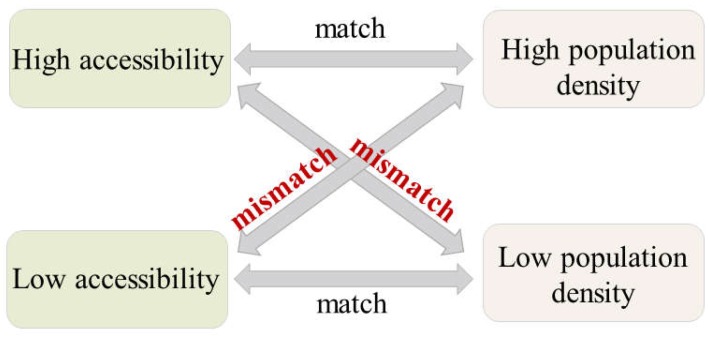
Distribution balance measurement of medical service facility accessibility.

The measurement of accessibility of medical service facilities is usually considered a valid method for evaluating medical service distribution in urban regions. Nevertheless, the accessibility measurement reflects merely a synthesis of the capacity and demand for a medical service, which is not sufficient to indicate the spatial equity of the medical service facility. As previous research insisted, public facilities can be considered equally distributed when the accessibility level in the city matches the population distribution level, meaning high accessibility should serve high population density regions and vice versa [41,42]. This is because the demand for medical service facilities is always from the residents, and the residents’ distribution can be measured by the population density. Yet the population distribution level and public facility accessibility are not quantitatively compared in most previous researches. A comparison system is designed based on Z-score normalization to measure the match degree between medical service facility accessibilities and the population distribution level in the city. The Z-score normalization method is able to normalize a set of values varying from below zero to more than zero. Here, in this research, we define the assessment as high when the value is positive (above zero) and define the assessment as low when the value is negative (below zero) for both normalized measurements of accessibility and population density. For each spatial unit, when high accessibility of medical service facilities is accompanied by high population density or low accessibility is accompanied by low population density, the distribution of medical service accessibility in this area is relatively balanced. On the other hand, when the area suffers from low accessibility of medical service facilities with high population density, or bears high accessibility with low population density, the medical service facility distribution is not balanced in the area. This is because high population density generates high demand for medical service accessibility, otherwise, low facility accessibility might be insufficient for highly-concentrated residents. When an area has a low population density, high accessibility would be a waste for lowly-concentrated residents. For example, if an area has a relatively low population density with a large-capacity municipal hospital, the scale of the hospital exceeds expectations leading to a waste of medical resources, and the low population could hardly support the big hospital’s operation. The match and mismatch discrimination method is depicted in Figure 5.

## 4. Results and Discussion

The methodology described in Section 3 is applied to the real Shenzhen dataset. First, the accessibilities of the three levels of medical service facilities in Shenzhen are computed applying the method of H2SFCA. Then, based on the values of accessibility, the distribution balance for each level of medical facility is calculated. Finally, balanced evaluations of the three levels of medical service facility accessibility are synthesized, and an integrated view of medical service facility balance is obtained.

### 4.1. Results of the Accessibility Evaluation

#### 4.1.1. Municipal Hospital Accessibility

**Figure 6:**
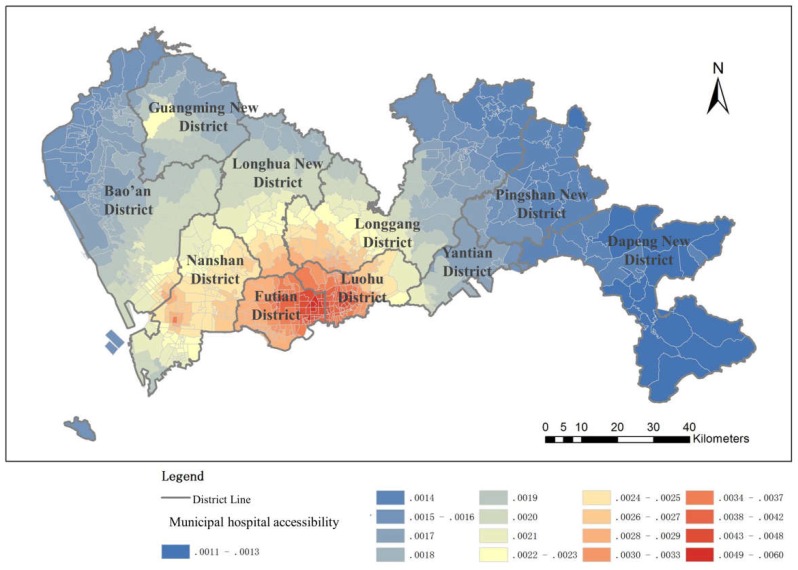
Municipal hospital accessibility

The results of the municipal hospital accessibility analysis show a clear outward decentering of the center-level structure; the accessibility is better within the SEZ than it is outside the SEZ, where it is relatively poor (Figure 6). There exists a large gap between accessibility values in the two regions. The municipal hospitals in Shenzhen are mostly distributed inside the SEZ to provide the residents living inside the SEZ with better accessibility, especially in the region near the city center, while there are few municipal hospitals outside the SEZ, leading to lower accessibility. Even after the normalization of the accessibility of medical facilities inside and outside the SEZ, the uneven distribution of medical resources is still obvious, despite the construction of a new tertiary hospital in the northwest region, which mitigates the enormous difference. In the early development of Shenzhen, the inner SEZ region was the most developed area, and thus, the higher-level medical service facilities are mostly distributed inside the SEZ. As a result, municipal hospital accessibility decays from the city center to the regions outside the SEZ. Although efforts have been made to improve municipal-level public services outside the SEZ, more time is required to overcome the imbalanced public service situation in the city.

#### 4.1.2. Town-Level Hospital Accessibility

**Figure 7:**
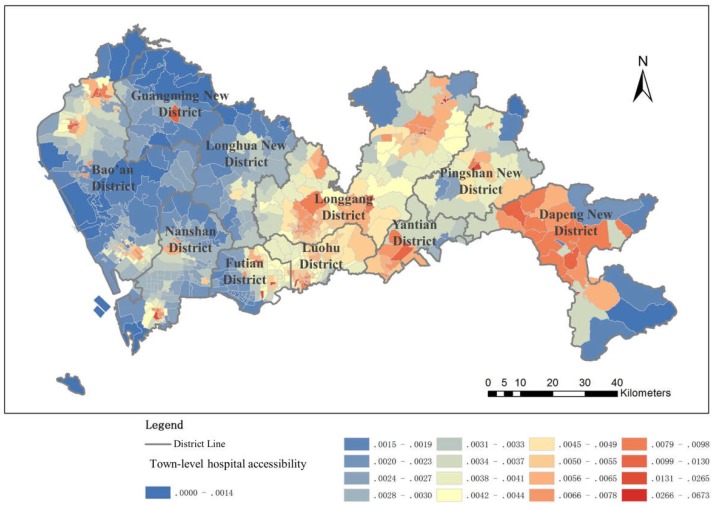
Town-level hospital accessibility

The town-level hospital accessibility distribution seems better than the municipal hospital accessibility distribution, especially in the area inside the SEZ and in the northeast area, while in large areas of the western region, medical accessibility is not as common (Figure 7). This is because the town-level hospitals of Shenzhen are more widespread in the city center and in the early developed regions (located mostly in the northeast), and are less widespread in the western part of Shenzhen. There are also relatively few town-level hospitals in the eastern area, yet due to the relatively small population, medical resources are fairly well distributed. Therefore, the town-level hospital accessibility is relatively good.

#### 4.1.3. Community-Level Hospital Accessibility

**Figure 8:**
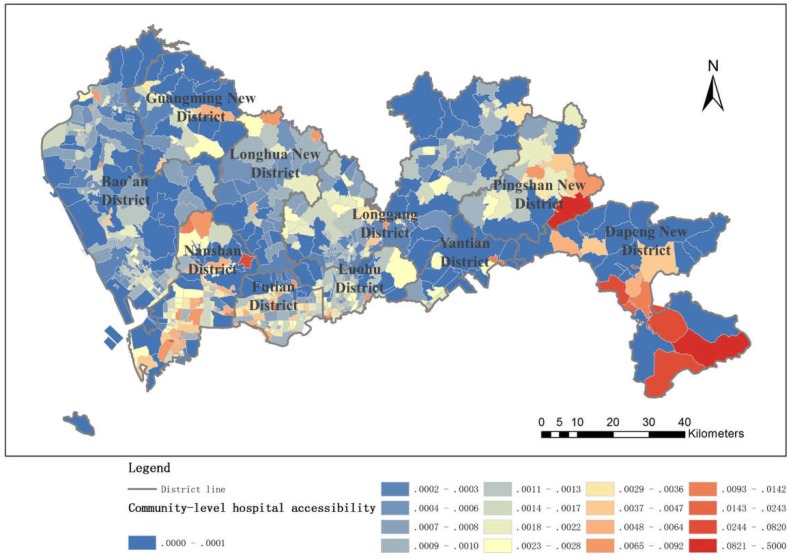
Community-level hospital accessibility.

Community-level hospital accessibility is generally well distributed, and outside the SEZ, there is even a slight advantage. This is because grassroots community health services in Shenzhen City have received more attention than in other areas; each neighborhood has established a community health service center, leading to more balanced community health accessibility. The distribution of community health service centers in Shenzhen is relatively uniform, and this convenience allows residents to go to community hospitals for medical services for minor illnesses (Figure 8).

#### 4.1.4. General Hospital Accessibility

**Figure 9:**
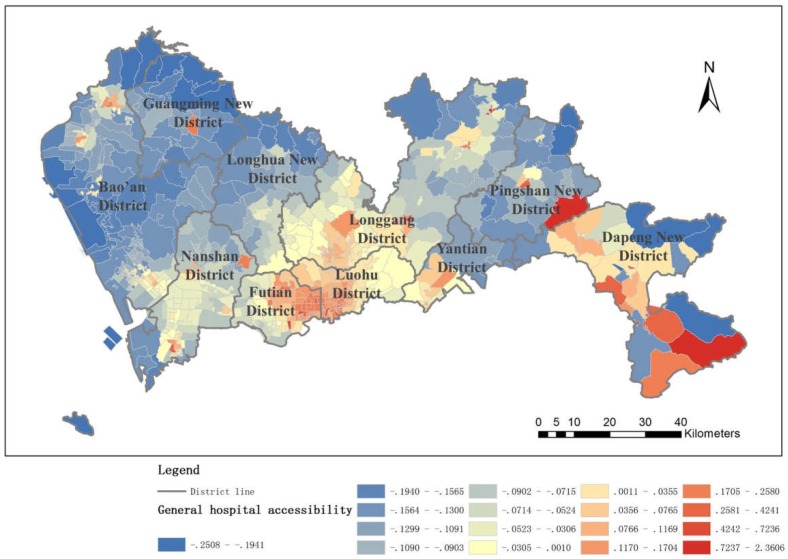
General hospital accessibility.

An integrated view of public health service accessibility in the whole city is synthesized by the three-level hospital accessibilities along with their weights (Figure 9). It is clearly demonstrated that the accessibility to public health services in Shenzhen is distributed in spatial clusters with a strong dependency on distance to the hospitals. As the distance to the hospital decays, the accessibility also decays. Generally, the area inside the SEZ bears large scales of aggregation. Specifically, in the northern region of the SEZ, the speed of decay appears slower than that of the eastern and western regions. However, accessibility in the area outside the SEZ is centralized in small regions where hospitals are located. The accessibility inside the SEZ is significantly better than outside the SEZ, and the accessibility is greater in the eastern part of the city than in the western part.

In particular, Futian District and Luohu District, which are located inside the SEZ, have achieved a remarkable mark of public health service accessibility. Especially in Futian District, high accessibility regions overlap all regions with high density populations. This phenomenon might be caused by the rapid economic growth of those two districts, and thus, medical services at all levels are concentrated in the region. On the other hand, the regions outside the SEZ suffer from low accessibility to public health services on average because the population distribution and the medical service distribution do not match, leading to a severely competitive situation for residents who to go to the hospitals. There are exceptions, such as the part of Longgang District close to the SEZ and Longhua New District. In those areas near the SEZ, the influence of good economic conditions and well-designed facilities has extended accessibility to a certain degree.

As illustrated in the results, analyses that consider population distribution are indispensable. Therefore, it is essential to further discuss the relationship between population distribution and the accessibility and balance of public service distribution.

### 4.2. Result of the Distribution Balance Evaluation

As discussed in the results of the accessibility evaluation for the three medical service levels, the population density distribution has a close connection with the distribution of accessibility. The balance of the accessibility distribution of medical service facilities is not indicated by the distribution of accessibility, as demonstrated in the previous subsection.

By utilizing the measurement of distribution balance, which combines normalized accessibility and population density, the results of the balance evaluation of the medical service accessibility are evaluated as follows.

#### 4.2.1. Municipal Hospital Accessibility Distribution Balance

**Figure 10:**
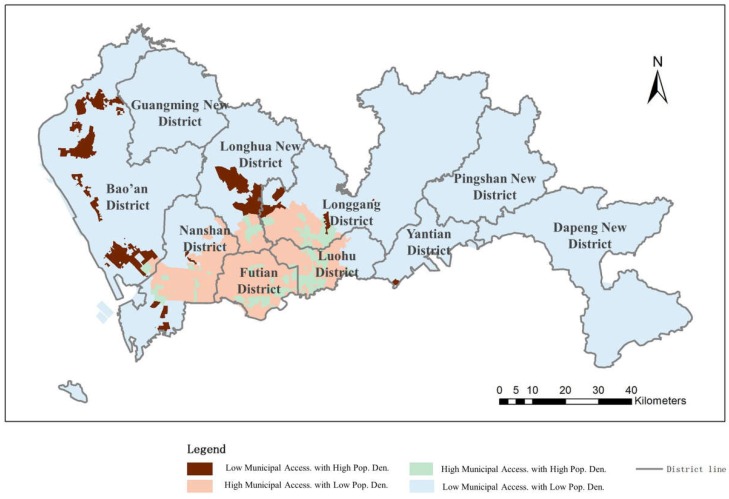
Balance evaluation between municipal hospital accessibility and population density.

The correlation between population density and the accessibility of municipal hospitals is shown in Figure 10. As introduced previously, we classified the matching conditions into four categories. The results show that 2.69% of the urban region retains high accessibility along with high population density, indicating a good balance in accessibility distribution. This area comprises 18.97% of the urban population and is mostly located inside the SEZ due to sufficient municipal medical facilities and concentrated population distribution. Approximately 83.79% of the region has a low population density along with low medical service facility accessibility; this area is mainly located outside of the SEZ, with 53.79% of the population in Shenzhen City. In contrast, 10.06% of the urban area possesses high accessibility with a low population density and is distributed mainly inside the SEZ. This area includes 10.91% of the population in Shenzhen. For those people, medical facilities are in surplus for a relatively low population density. However, the remaining 3.45% of the area in Shenzhen suffers from low accessibility with a high population density, mostly outside the SEZ. In such regions, the resident distribution is dense (with 16.34% residents living inside these regions), yet medical service facilities are extremely limited.

Overall, municipal hospital accessibility inside the SEZ is sufficient and even extends beyond demand in some areas. In total, 70% of the residents in Shenzhen are enjoying balanced accessibility. The rest of the residents in Shenzhen mostly live outside the SEZ and suffer from insufficient municipal medical facilities, especially in regions with large population densities.

#### 4.2.2. Town-Level Hospital Accessibility Distribution Balance

**Figure 11:**
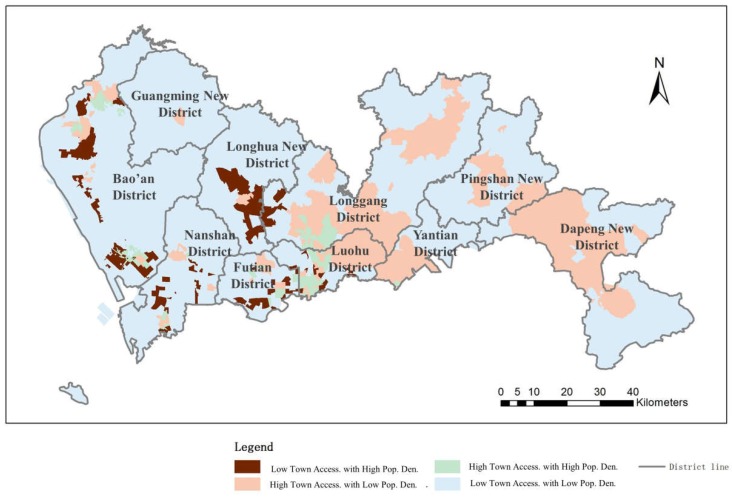
Balance evaluation between town-level hospital accessibility and population density.

The results of the balance evaluation between population density and accessibility of town-level hospitals are shown in Figure 11. The results show that 2.12% of the region and 14.52% of the population of the whole city have high accessibility to medical service facilities along with a high population density. These regions are mainly located inside the SEZ and in the mid-north of the city. Approximately 73.41% of the urban area has low medical accessibility and a low population density, consisting of 48% of the population in total. These regions are distributed sparsely throughout the whole urban region but mainly outside the SEZ. In these areas, the population density level corresponds with the level of medical services in the city, indicating a balanced distribution of medical service facilities. On the other hand, 20.44% of urban areas possess high accessibility of medical services and comprise a low population density of 16.69% residents in Shenzhen; this area is mainly concentrated inside the SEZ. Medical facilities exceed demand in these regions, and the population and the medical service facilities are not distributed in a balanced way. Regions covering 4.02% of the urban area bear low medical service accessibility and include a high population of 20.79% of all city residents. In such regions, limited medical service facilities cannot support the large number of people living in a small area.

Generally speaking, 55.4% of residents in Shenzhen have appropriate accessibility to town-level hospitals; these are residents who mainly live inside the SEZ or who are dispersed in low population areas. The distribution of population and town-level hospitals is relatively balanced for over half of the population in Shenzhen. The rest of the residents are living in areas suffering from an unbalanced distribution of population and town-level hospitals; these areas have either redundant or insufficient facilities.

#### 4.2.3. Community-Level Hospital Accessibility Distribution Balance

**Figure 12:**
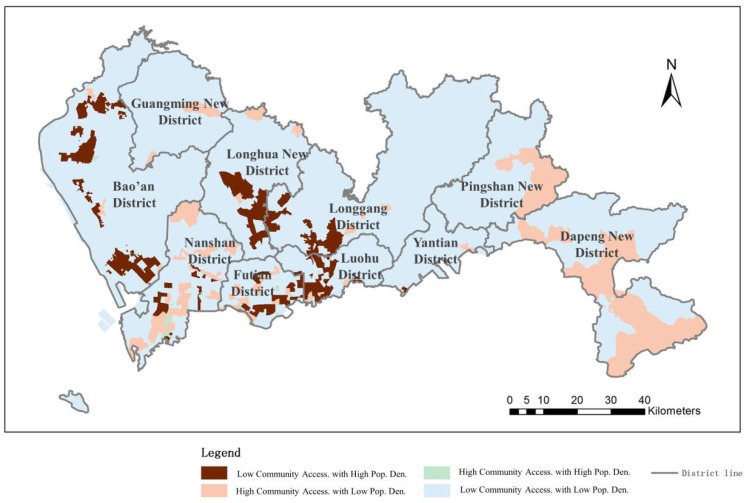
Balance evaluation between community-level hospital accessibility and population density.

The results of the balance evaluation between population density and the accessibility of community-level hospitals are shown in Figure 12. The results show that 2.92% of the city’s regions have high community-level hospital accessibility and a high population density (15.29% of the population); these areas are mainly located inside the SEZ. Approximately 72.25% of the urban region has a low population density (1.53% of the population of Shenzhen) and low community-level service accessibility; these areas are located citywide but are less common inside the SEZ. In these areas, the distribution of community-level hospitals and the population density are balanced. Nevertheless, the rest of the city suffers from an unbalanced distribution. A total of 11.64% of the city’s area, with 3.26% of the residents, has high community-level hospital accessibility with a low population density and uniform distribution throughout the city. The number of community-level hospitals in these areas has exceeded the demands of the residents. In contrast, 5.85% of the area, which contains 33.78% of the residents, suffers from low community-level health care facilities combined with high population densities.

The results indicate that even though the distribution of community hospital accessibility alone seems balanced in Shenzhen (Figure 7), over half of the residents suffer from an unbalanced distribution of community-level hospitals with respect to population density, especially in areas that contain a large proportion of the city’s population. The distribution of community hospital accessibility in such areas mainly associates with the size of the area instead of the population density.

#### 4.2.4. General Hospital Accessibility Distribution Balance

**Figure 13:**
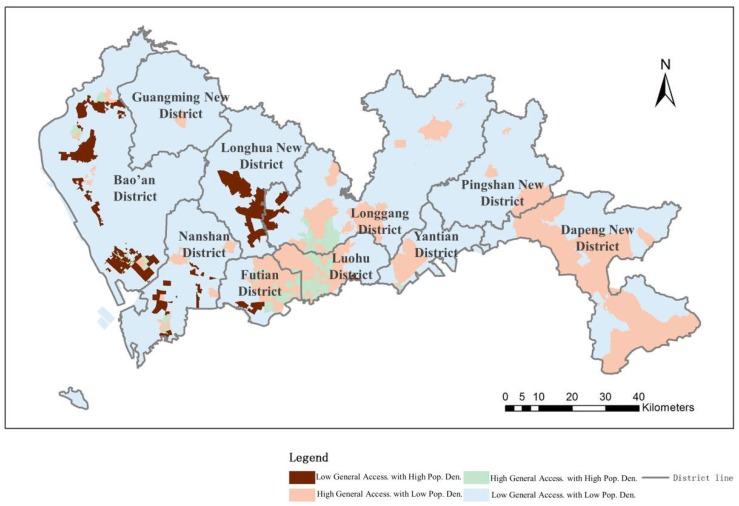
Balance evaluation between all medical service facility accessibility and population density levels.

Additionally, a synthesis of all three levels of medical service accessibility is obtained from the population data in Shenzhen to evaluate the distribution balance of medical service facilities in an integrated way. From the results shown in Figure 13, 79.57% of the urban area, which contains 69.61% of the population, has a balanced distribution of medical facility accessibility and population. This finding means that the accessibility level and the population density level in the city are consistent with each other for 69.61% of residents, while for the rest of the residents, the distribution of medical service accessibility is not balanced, especially for people living in areas with a high population density and low hospital accessibility. The high accessibility and low population density regions are located mainly in the city center and in the northeast region and include only 10.83% of the total number of residents. The phenomenon of excess medical facilities is not common in Shenzhen. However, the distribution of the medical service facilities is critically unbalanced in general because 19.65% of the residents in Shenzhen suffer from low medical service accessibility and a high population density. In total, over 30% of the residents are enduring an unbalanced distribution of medical service accessibility.

## 5. Conclusions

Public medical services are designed and built for diverse treatment purposes, which can be categorized into multiple levels. Although abundant researches have focused on public medical service accessibilities, the medical service level issue is often neglected. In this research, considering that different levels of medical service facilities in the city have different service scopes, we focused on constructing a framework for the measurement of the accessibility of three levels of public medical service facilities from a hierarchical perspective, along with a hierarchical evaluation of medical service facility distribution equity. Two major contributions were achieved in this study. First, considering that the treatment scope of public medical services varies with the medical service facility level, we proposed a hierarchical measurement of accessibility, H2SFCA, with several distance attenuation functions corresponding to the service scopes of the three levels of medical service facilities, in order to obtain a more accurate and comprehensive medical accessibility measurement.

Thus, a fresh view of hierarchy was brought to the facility accessibility assessment issue, ameliorating the accessibility measuring methods. Second, an evaluation method for medical service facility distribution balance was proposed by comparing the accessibility level and population density level in the city. This contributes to the evaluation of public facility distribution equilibrium in general.

The metropolis of Shenzhen was chosen as the study area, and real datasets from Shenzhen City were applied to evaluate the accessibility of medical service facilities and the balance of medical service distribution. According to the results and discussions from this empirical study, which utilizes Shenzhen medical service facility data and population data, several issues with medical service distribution balancing are revealed. First, the distribution of medical service facilities varies with different levels of medical service. For example, in Shenzhen, even though the SEZ regions generally have better medical accessibility than areas outside the SEZ regions, high accessibility to municipal hospitals is concentrated inside the SEZ, while lower-level hospitals have fewer accessibility distribution differences. The proposed H2SFCA method has proved its advantages as a comprehensive medical service accessibility measurement at different medical service levels. Second, the equity of medical service facility accessibility does not necessarily depend on accessibility distribution alone. Population distribution should also be taken into account when discussing the balance of service facility distribution. We located certain regions suffering from an unbalanced distribution of medical service facilities in Shenzhen, where residents could not obtain convenient health care services. Overall, we found that Shenzhen is currently suffering from a critical issue in medical service facility distribution and that more than 30% of residents cannot benefit from the medical service distribution balance. City planners should focus more on the high-density districts outside the SEZ and improve medical service conditions by increasing the number of medical service facilities, especially municipal hospitals, in those areas.

Future work will focus on combining large datasets to validate and apply the multilevel hospital accessibility framework to multiple cities proposed in this research. The availability of mass spatial temporal data offers a major opportunity to evaluate the characteristics of public facilities, including medical services [43,44]. Further research on detailed medical service distributions and utilities should be performed next. Founded on the well-developed existing work on public transit with big data [45,46], the accessibility of other types of public facilities, such as transport facilities, can also be examined using the methods in this study. Additionally, when data from multiple cities are available, a comparison study between cities will be performed, and the patterns of medical service facility accessibility and balance should be discovered.

## Figures and Tables

**Table 1:** Table for distance decay indicators for different hospital levels.

Types of Medical Service Facilities	Transportation Mode	Truncation Distance	Attenuation Coefficient
Community-level hospitals	Walk	1000 m	2
Town-level hospitals	Vehicle travel	10,000 m	1
Municipal hospitals	Vehicle travel	Unlimited	0.5
